# Learning Outcomes of Fourth-Generation Total Wrist Arthroplasty Using the Motec Implant: Does Dual-Consultant Operating Improve Outcomes?

**DOI:** 10.7759/cureus.99754

**Published:** 2025-12-21

**Authors:** Siddhartha Murhekar, Sunandan Datta, Prashant Awasthi, Darren Leong, Ahmed Elkohail, Ali Soffar, Qazi Masood, Amit K Yadav, Sultan Alsayeh, Andrew Smith

**Affiliations:** 1 Trauma and Orthopaedics, Medway NHS Foundation Trust, Gillingham, GBR; 2 Trauma and Orthopaedics, Aneurin Bevan University Health Board, Newport, GBR; 3 Trauma and Orthopaedic Surgery, Queen Elizabeth The Queen Mother Hospital, Margate, GBR; 4 Trauma and Orthopaedics, Medway Maritime Hospital, Gillingham, GBR; 5 Trauma and Orthopaedics, Princess Royal University Hospital, King's College Hospital NHS Foundation Trust, London, GBR; 6 Trauma and Orthopaedics, King's College Hospital NHS Foundation Trust, London, GBR; 7 Orthopaedics and Traumatology, Grant Government Medical College and Sir J.J. Group of Hospitals, Mumbai, IND; 8 Hand Surgery, East Kent Hospitals University NHS Foundation Trust, Canterbury, GBR

**Keywords:** dual-consultant operating, learning curve, motec wrist prosthesis, total wrist arthroplasty, wrist arthritis

## Abstract

Introduction: Total wrist arthroplasty (TWA) has evolved as a motion-preserving alternative to arthrodesis, offering improved pain relief, functional outcomes, and patient satisfaction. The fourth-generation Motec wrist prosthesis, a cementless, ball-and-socket, metal-on-metal implant, has demonstrated encouraging mid- to long-term results. However, TWA remains a technically demanding, low-volume procedure with a steep learning curve. Dual-consultant operating has been proposed as a method to optimize learning outcomes and reduce complications, but evidence remains limited.

Methods: A retrospective service evaluation was conducted on all Motec TWA procedures performed between September 2021 and November 2024 by six fellowship-trained hand surgeons at Kent and Canterbury Hospital. Nineteen patients met the inclusion criteria and were divided into dual-consultant (n = 14) and single-consultant (n = 5) groups. Complications were classified according to the Clavien-Dindo system, and surgeon expertise was categorized per the Tang and Giddins classification. Statistical analysis was performed using Fisher’s exact test.

Results: Nineteen patients were evaluated, including 14 operated by dual consultants and 5 by a single consultant. In the dual-consultant group, 7 patients (50%) had no complications, 4 (28.6%) had Grade I, and 3 (21.4%) had Grade II complications. In the single-consultant group, 2 patients (40%) had no complications, 2 (40%) had Grade I, and 1 (20%) had a Grade IV complication.

Conclusion: Dual-consultant operating did not confer a statistically significant reduction in complications; however, an absence of severe complications in dual cases suggests potential benefits in safety and mentorship. Given the small sample size and high baseline expertise of surgeons, larger, prospective, multicenter studies are warranted to define the role of dual-consultant surgery in TWA.

## Introduction

Wrist joint disorders, including arthritis and trauma, can significantly impair hand function and quality of life. Traditional treatments often involve conservative management or fusion procedures, which, while effective in pain relief, can severely limit wrist mobility. Total wrist arthroplasty (TWA) has emerged as a motion-preserving alternative to wrist fusion for patients with advanced wrist arthritis, offering better functional outcomes and higher patient satisfaction compared to wrist arthrodesis. Recent advancements in design and surgical techniques have renewed interest in TWA [1].

Initially intended for older patients with inflammatory arthritis and lower functional demands, TWA is increasingly considered an alternative to primary wrist fusion in younger, more active patients who wish to maintain wrist motion [2]. Among the modern implants available, the Motec Wrist Joint Prosthesis represents a fourth-generation, cementless, ball-and-socket metal-on-metal design. It utilizes primary screw fixation with allowance for secondary bony ingrowth and is derived from the earlier bi-axial wrist replacement model, originally introduced in 2006 as the “Gibbon.” The implant was rebranded as the Motec in 2010 without design changes [2].

The clinical studies of the Motec wrist prosthesis have shown promising results, including pain relief, improvement in range of motion, and patient satisfaction, and have been a promising option for individuals seeking to regain independence and improvement in quality of life. Owing to being a low-volume surgery, TWA has been a novel technique in comparison to high-volume hip and knee replacements [3]. The peculiar aspects involving this novel technique are associated with a steep learning curve, and studies have shown that surgeons encounter technical and surgical complications who have limited experience in Motec wrist arthroplasty [4]. 

In the early adoption phase of newer techniques, improving the learning curve is important to reduce patient risk. Methods to improve the curve involve structured mentorship and dual-consultant operations. Among various methods, dual-consultant operating has shown promising results, especially in complex revisions, to reduce intraoperative blood loss, shorten operative time, and decrease inpatient hospital stay for patients [3]. However, there is a scarcity of evidence specifically assessing the effect of dual-consultant operating in TWA.

The study aims to assess the outcomes of TWA using Motec in terms of function, complications, and the impact of dual- versus single-consultant operating on the learning curves.

## Materials and methods

A retrospective, non-interventional service evaluation was conducted to assess patient outcomes following Motec TWA procedures performed between September 2021 and November 2024 by six fellowship-trained hand surgeons at the Kent and Canterbury Hospital.

Inclusion criteria

All patients who underwent Motec TWA during the study period and had a minimum follow-up of at least one month by November 20, 2024, were included in the analysis.

Exclusion criteria

Patients were excluded if they had undergone a non-Motec TWA, a primary or secondary wrist fusion procedure, or if they lacked postoperative follow-up or were scheduled only for a future surgery.

Patient data were anonymized and extracted from electronic patient records and medical files into Microsoft Excel (Microsoft Corp., Redmond, WA, USA). The procedures were listed chronologically (case numbers 1-23), with three cases excluded due to lack of follow-up, and one patient died due to small bowel obstruction, not related to the TWA, leaving a total study population of 19 patients. The follow-up of patients was calculated from the date of the index operation to a fixed review date, i.e., November 15, 2024, with a minimum, maximum, and mean follow-up calculated accordingly.

The complications of procedures were classified into six further groups, i.e., Grade 0 to Grade V, based on the consequences they have for the patient, requirement for a revision surgery, and survival of the implant [5]. Statistical analysis was performed using IBM SPSS Statistics for Windows, Version 29.0 (Released 2022; IBM Corp., Armonk, NY, USA). Fisher’s exact test was used for comparison between groups, and the level of surgeon expertise was classified according to the Tang and Giddins classification [6].

## Results

Nineteen patients were evaluated in this study, divided into two groups based on the involvement of surgical consultants: 14 cases were operated on by dual consultants, and 5 were operated on by a single consultant (Table 1).

**Table 1:** Comparison of postoperative complication grades between dual-consultant and single-consultant cases. Complications are categorized from Grade 0 to Grade V based on the impact they have on the patient, the requirement of revision surgery, and survival of the implant.

Group	No complication	Grade I	Grade II	Grade IV	Total patients
Dual consultant	7 (50%)	4 (28.6%)	3 (21.4%)	0 (0%)	14
Single consultant	2 (40%)	2 (40%)	0 (0%)	1 (20%)	5

In the dual-consultant group, seven patients (50%) experienced no postoperative complications. Four patients (28.6%) developed Grade I complications, defined as minor deviations from the normal postoperative course, requiring no intervention beyond analgesics or antiemetics. Three patients (21.4%) experienced Grade II complications, all of which were iatrogenic extensor pollicis longus tendon ruptures identified intraoperatively. These were managed with immediate tendon repair during the index procedure, followed by structured postoperative hand therapy focusing on strengthening and rehabilitation of the repaired tendon.

In contrast, within the single-consultant operating group, two patients (40%) experienced no postoperative complications. Two patients (40%) experienced Grade I complications. One patient (20%) developed a Grade IV complication consisting of a fracture of the metacarpal component, necessitating revision surgery with replacement of the fixation device while retaining the radial component of the arthroplasty.

A statistical comparison of complication rates using Fisher’s exact test [7] between the two groups showed no significant difference (p = 1), suggesting comparable complication incidences between the two groups in this limited cohort.

## Discussion

TWA has advanced significantly over the recent years, with fourth-generation implants, such as the Motec system, showing improved outcomes in terms of better range of motion, patient satisfaction, and better design compared to earlier generations [8,9]. Recent studies have demonstrated the longevity of the prosthesis, with Reigstad et al. reporting an 86% survival rate for the Motec wrist at 10 years [2].

Although originally designed for low-demand patients with inflammatory arthritis, TWA is increasingly being used for younger, more active individuals seeking to maintain wrist function [8]. Careful patient selection with individual expectations and needs is paramount before offering TWA to patients [10]. Although there was no significant difference between the Disabilities of the Arm, Shoulder and Hand (DASH) inventory, and the Patient-Rated Wrist Evaluation (PRWE) score for TWA versus wrist arthrodesis, there was a positive trend in terms of personal hygiene and fastening buttons in patients with TWA [11]. Satisfaction was found to be similar in both arthrodesis and arthroplasty groups [12].

It is essential to understand the learning curve associated with newer implant systems like the Motec, as it is being used in increasingly younger patients with a more active lifestyle. One article studies the impact of the learning curve on optimizing surgical outcomes and patient satisfaction [11].

As with other joint replacements that have a steep learning curve, outcomes are closely associated with the surgical team’s experience, as highlighted in the review by Sarpong et al. [13]. The research by Tang and Giddins also underscores the critical role of surgical experience in reducing technical errors and optimizing patient outcomes [6]. Performing the surgery with dual consultants may provide educational value for less experienced surgeons by offering real-time guidance and support in decision-making. It is worth noting that all six hand surgeons involved in these 20 cases were fellowship-trained in TWA. According to the Tang and Giddins classification, all our surgeons are categorized as Level 3 or 4 [6].

The primary aim of our study was to investigate whether dual-consultant operating improves outcomes in Motec wrist arthroplasty, particularly in the context of a low-volume, high-complexity procedure. Our retrospective analysis of 19 patients operated on by six consultant hand surgeons focused on evaluating whether consultant team composition influenced postoperative results.

We found no statistically significant difference in complication rates between single- and dual-consultant procedures. Although the dual-consultant group had a higher proportion of patients without complications (50% vs. 40%) and fewer severe complications (with no Grade IV events), the p-value (1.0) indicates that these differences are likely due to chance rather than a true effect of operating with two consultants.

This contrasts sharply with the findings of Brown et al. [4], who conducted a cohort study of 30 patients treated by six experienced hand surgeons performing their first 30 Motec TWAs.

These findings need to be studied with caution. It is plausible that our surgeons had already independently surpassed the initial, steep phase of the learning curve, the 15- to 30-case threshold identified, before the period of this retrospective study. Consequently, our study may not be capturing the learning phase at all, but rather the performance of surgeons who have achieved proficiency. This would inherently lead to a lower and more stable complication rate across the board, masking any potential benefits of a dual-consultant approach that would be more apparent during the initial learning period.

Another important consideration is the statistical power of our study. With a small sample size of 19 patients, our analysis is likely underpowered to detect a subtle, yet clinically meaningful, difference between the two groups. The observed trend of fewer complications in the dual-consultant group might indeed be a true effect that would become statistically significant in a larger cohort. The p-value of 1.0, while indicating no difference, must be interpreted with caution in the context of this limitation. A larger, prospective study would be necessary to definitively assess the value of dual consultant surgery in this setting.

Finally, we must understand the evolution of the surgical technique itself, what could be termed the "implant's learning curve." The hand surgeons in our study are operating at a later point in time than the early adopters [4]. Over the years, collective knowledge about the Motec system has improved, leading to refined techniques for soft tissue balancing, optimal component positioning, and preventing impingement [14]. Our cohort benefits from this mature understanding, which was not available to the very first surgeons adopting the prosthesis. Therefore, the baseline standard of care has improved, contributing to better outcomes for all patients, irrespective of the number of hand surgeons present.

A primary strength of this study is its multi-surgeon, regional cohort design. By including all Motec TWA procedures performed by six specialist hand surgeons across the KSS region, the findings possess greater external validity than a single-surgeon series and are more representative of a regional standard of care. The methodological rigor is enhanced by the use of standardized, validated classification systems. Employing the Redfern framework for complications and the Tang and Giddins classification for surgeon experience ensures that our data is objective, reproducible, and directly comparable to other key studies in the field. This systematic approach to data collection provides a robust foundation for evaluating outcomes in this complex, low-volume procedure.

The principal limitation of our study is its retrospective nature combined with a small sample size. With only 19 patients included for analysis, the study is significantly underpowered to detect a true difference in outcomes between single- and dual-consultant procedures, as reflected in the statistically nonsignificant result. This is particularly evident in the imbalanced comparison groups of 5 single-consultant and 14 dual-consultant cases. Furthermore, the retrospective design leads to variable follow-up durations, which may not be sufficient to capture long-term complications, such as component loosening or late-onset osteolysis. Consequently, while our findings are valuable for generating hypotheses, they should be interpreted with caution, and a larger, prospective multicenter study is required to definitively assess the impact of dual-consultant operating on TWA outcomes.

## Conclusions

This study revealed that there was no statistically significant benefit in dual-consultant operating versus a single-surgeon approach in TWA after reviewing postoperative complications. One of the reasons for this is the high level of surgical expertise among the six surgeons who were fellowship-trained hand surgeons and had gone beyond the initial steep learning curve. Remarkably, the complete absence of severe complications in dual-consultant operating hints at a potential, albeit unproven, safety advantage. The main limitation of the study was a small sample size, which lacks statistical power. TWA is a low-volume surgery, and hence, finding a bigger sample size would need multicenter coordination or international cohorts. Our study findings suggest that dual-consultant operating may not be required for surgeons with expertise, although the true value may lie in training and mentorship during a surgeon’s early experience. A prospective, multicenter study is required to clarify the role of dual-consultant operating and to establish best practices for optimizing patient safety in this demanding procedure.
